# Supplementary material for: FastqPuri: high-performance preprocessing of RNA-seq data
Source: BMC Bioinformatics. 2019 May 3;20:226. doi: 10.1186/s12859-019-2799-0 (PMC6500068; doi:10.1186/s12859-019-2799-0)
Supplement: Supplementary file 2 — Archive of FastqPuri. Archive containing all files needed to install and run FastqPuri v1.0.6. Date stamp March 22, 2019. (GZ 47,819 kb) [file 12859_2019_2799_MOESM2_ESM.gz › FastqPuri-1.0.6/html/globals_b.html]

FastqPuri: File Members


FastqPuri


Here is a list of all documented file members with links to the documentation:

### - b -

- B\_LEN
  : defines.h
- BASESPERCHAR
  : defines.h
- belongsto()
  : stats\_info.c
- Bfilter
  : bloom.h
- Bfkmer
  : bloom.h
- bitMask
  : bloom.c
- BITSPERCHAR
  : defines.h
- BLOOM
  : defines.h
- bool
  : defines.h
- buffer\_output()
  : io\_trimFilter.c
  , io\_trimFilter.h
- buffer\_outputDS()
  : io\_trimFilterDS.h
- bw\_1B
  : Lmer.c
  , trimDS.c
  , adapters.c


---

Generated on Mon Mar 19 2018 23:42:01 for FastqPuri by  

 1.8.14
